# Signal Strength as an Important Factor in the Analysis of Peripapillary Microvascular Density Using Optical Coherence Tomography Angiography

**DOI:** 10.1038/s41598-019-52818-x

**Published:** 2019-11-08

**Authors:** Hyung Bin Lim, Yong Woo Kim, Ki Yup Nam, Cheon Kuk Ryu, Young Joon Jo, Jung Yeul Kim

**Affiliations:** 1 Department of Ophthalmology, Chungnam National University College of Medicine, Daejeon, Republic of Korea; 2 Department of Ophthalmology, Armed Forces Capital Hospital, Seongnam, Republic of Korea; 3 Department of Ophthalmology, Seoul National University College of Medicine, Seoul, Republic of Korea; 4 Department of Ophthalmology, Gyeongsang National University Changwon Hospital, Changwon, Republic of Korea

**Keywords:** Optic nerve diseases, Eye manifestations, Medical imaging

## Abstract

The quality of the scan image is important in peripapillary circulation analysis using optical coherence tomography angiography (OCTA). We aimed to investigate the effects of signal strength (SS) on the peripapillary microvascular density acquired from OCTA. A total of 259 eyes from 259 young healthy subjects were included. Peripapillary OCTA images using 3 × 3 mm angiography scan were acquired from all participants. Subjects were divided into four groups according to the SS: SS 7, SS 8, SS 9, and SS 10. Vessel density (VD) and perfusion density (PD) of the superficial capillary plexus were calculated. VD and PD were compared among the four groups, and linear regression analyses were performed to identify and evaluate the clinical factors associated with average VD. As the SS increased from 7 to 10, the average VD and PD increased; these increases were statistically significant (all, p < 0.001). Regression analyses showed that four factors were significantly correlated with average VD: age (partial r = 0.133), average retinal nerve fiber layer thickness (partial r = 0.169), cup/disc ratio (partial r =−0.481), and SS (partial r = 0.413). SS is a significant factor affecting peripapillary microvascular density, and its influence is similar to well-known structural parameters associated with glaucoma.

## Introduction

Ocular blood flow is thought to have an important role in various types of optic neuropathies including glaucoma, ischemic optic neuropathy, and hereditary optic neuropathy^1^. Although various imaging modalities such as fluorescein angiography or indocyanine green angiography^2,3^, magnetic resonance imaging^4^, ultrasound color Doppler imaging^5^, and laser Doppler flowmetry^6^ have been used for ocular blood flow analyses, these techniques are not widely used in clinical practice, due to various limitations.

Optical coherence tomography angiography (OCTA), recently introduced, provides qualitative and quantitative information that had not been previously available. Due to its fast, noninvasive assessment of the capillary network, OCTA is commonly used in the diagnosis and management of retinal and optic disc diseases^7,8^. Previous OCTA studies have reported that peripapillary vessel density (VD) in glaucomatous eyes is significantly reduced compared to that in healthy eyes and as such, has been proposed as a diagnostic indicator of glaucoma^9–19^. It also has been reported that peripapillary VD measurements in Leber’s hereditary optic neuropathy (LHON)^20,21^ or optic neuritis (ON)^22^ can be used as biomarkers.

Microvascular density is associated not only with the disease pathophysiology but also with other factors such as age^23^, axial length^24^, and image quality^25^. These factors can act as confounding factors and must be considered in the analyses of OCTA results. Previously, we confirmed that signal strength (SS) strongly influences OCTA measurements in macular OCTA analyses^26^. SS is also thought to play an important role in optic disc and peripapillary perfusion analyses. However, the reference level of the SS in the analyses of optic nerve head and peripapillary microvascular perfusion has yet to be determined. Because the vessel distributions of macular and peripapillary areas considerably differ, information on the effects of SS on OCTA measurements in the peripapillary area is required.

Therefore, we investigated the effects of SS on peripapillary microvascular density measured from OCTA to determine the appropriate level for peripapillary perfusion analyses.

## Results

### Patient characteristics

In total, 150 eyes of males and 109 eyes of females were enrolled. The number of eyes in each group was 99 (SS 7), 82 (SS 8), 42 (SS 9), and 36 (SS10). The mean ages of the SS 7, SS 8, SS 9, and SS 10 groups were 26.62 ± 8.55, 26.80 ± 8.38, 25.84 ± 6.71, and 27.56 ± 5.26 years, respectively. No statistically significant differences were found in age and sex among the four groups. There were no significant differences in ocular characteristics including the BCVA and spherical equivalent, IOP, central corneal thickness, axial length, central macular thickness, and RNFL thickness. The baseline characteristics of the participants are shown in Table 1.

**Table 1:** Demographics and clinical characteristics of the participants.

	SS 7 (n = 99)	SS 8 (n = 82)	SS 9 (n = 42)	SS 10 (n = 36)	p-value
Age (mean ± SD, years)	26.62 ± 8.55	26.80 ± 8.38	25.84 ± 6.71	27.56 ± 5.26	0.382^*^
Sex (male/female)	62/37	44/38	22/20	22/14	0.534^†^
BCVA (mean ± SD, LogMAR)	0.02 ± 0.06	0.02 ± 0.06	0.01 ± 0.03	0.01 ± 0.08	0.203^*^
Spherical equivalent (mean ± SD, diopters)	−2.39 ± 2.53	−2.06 ± 2.56	−2.75 ± 3.22	−3.93 ± 3.20	0.092^*^
IOP (mean ± SD, mmHg)	16.06 ± 3.40	15.28 ± 3.63	15.93 ± 2.91	16.33 ± 3.36	0.130^*^
Central corneal thickness (mean ± SD, μm)	548.98 ± 43.94	538.76 ± 48.96	539.52 ± 40.76	544.68 ± 47.31	0.234^*^
Axial length (mean ± SD, mm)	25.01 ± 1.29	25.15 ± 1.27	25.10 ± 1.61	25.63 ± 1.68	0.095^*^
Central macular thickness (mean ± SD, μm)	252.41 ± 20.40	253.40 ± 20.62	251.24 ± 20.36	261.17 ± 15.66	0.099^*^
Rim area (mean ± SD, mm2)	1.27 ± 0.23	1.28 ± 0.23	1.33 ± 0.23	1.35 ± 0.31	0.145^*^
Cup/disc ratio (mean ± SD)	0.48 ± 0.15	0.49 ± 0.17	0.44 ± 0.19	0.46 ± 0.16	0.102^*^
Average RNFL thickness (mean ± SD, μm)	95.65 ± 8.75	96.01 ± 8.68	96.51 ± 8.80	98.63 ± 9.11	0.317^*^
Superior sector (mean ± SD, μm)	120.90 ± 15.88	118.68 ± 16.57	120.54 ± 16.81	122.94 ± 15.97	0.500^*^
Temporal sector (mean ± SD, μm)	76.93 ± 14.96	77.15 ± 13.68	77.92 ± 15.60	83.14 ± 17.10	0.139^*^
Inferior sector (mean ± SD, μm)	119.62 ± 18.55	120.48 ± 17.12	120.00 ± 22.18	121.69 ± 18.37	0.928^*^
Nasal sector (mean ± SD, μm)	66.40 ± 11.58	65.63 ± 9.61	67.41 ± 12.91	66.83 ± 8.57	0.771^*^

SS = signal strength; SD = standard deviation; BCVA = best-corrected visual acuity; logMAR = logarithm of the minimum angle of resolutions; IOP = intraocular pressure; RNFL = retinal nerve fiber layer

^*^The *p*-value was obtained from one-way analysis of variance (ANOVA).

^†^The *p*-value was obtained from the chi-square test.

### Vessel density and perfusion density

**Figure 1:**
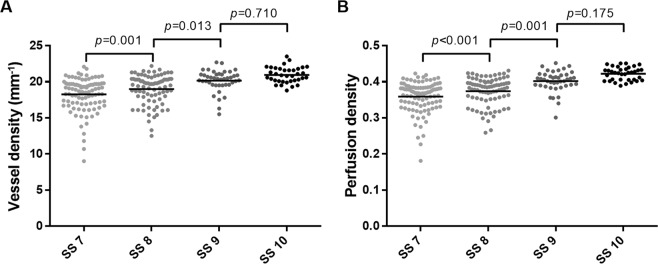
Scatter plots showing the significant relationships between SS and average VD (**A**) and average PD (**B**). The p-values were calculated using one-way analysis of variance, with a *post hoc* Bonferroni correction.

The average VD values of SS 7, SS 8, SS 9, and SS 10 groups were 18.06 ± 2.66, 18.98 ± 1.95, 20.16 ± 1.47, and 20.95 ± 1.09 mm^-1^, respectively (p < 0.001, Table 2, Fig. 1A). In *post hoc* analyses, there were significant differences among the SS 7, SS 8, and SS 9 groups (all, p < 0.05), whereas no significant differences were found between SS 9 and SS 10 groups (p = 0.710). In superior, temporal, and inferior sectors, SS 8, SS 9, and SS 10 groups showed no difference (all, p > 0.05), but were significantly higher than SS 7 (all, p < 0.05). On the other hand, the nasal sector showed a different pattern. As the SS increased from 7 to 10, the VD measurements tended to increase; these differences were statistically significant (all, p < 0.05). In the PD analyses, the average PD values of SS 7, SS 8, SS 9, and SS 10 groups were 0.356 ± 0.049, 0.375 ± 0.037, 0.402 ± 0.029, and 0.422 ± 0.018, respectively (p < 0.001, Table 3, Fig. 1B). All measurements including the average, superior, temporal, inferior, and nasal sectors, showed the same trends as those for VD.

**Table 2:** Comparison of the vessel density among groups with a signal strength (SS) from 7 to 10 (mm^−1^).

	SS 7 (n = 99)	SS 8 (n = 82)	SS 9 (n = 42)	SS 10 (n = 36)	p-value^*^	p-value^†^	p-value^‡^	p-value^§^
Average	18.06 ± 2.66	18.98 ± 1.95	20.16 ± 1.47	20.95 ± 1.09	**<0.001 (7 < 8 < 9 = 10)**	**0.001**	**0.013**	0.710
Superior sector	19.04 ± 2.50	19.63 ± 1.86	20.24 ± 1.70	20.28 ± 1.37	**<0.001 (7 < 8 = 9 = 10)**	**0.045**	0.606	1.000
Temporal sector	19.31 ± 3.71	20.80 ± 2.84	21.74 ± 2.30	21.41 ± 2.38	**<0.001 (7 < 8 = 9 = 10)**	**<0.001**	0.543	1.000
Inferior sector	19.04 ± 2.50	19.75 ± 1.53	20.25 ± 1.33	20.56 ± 1.36	**<0.001 (7 < 8 = 9 = 10)**	**0.005**	0.907	1.000
Nasal sector	15.18 ± 3.81	16.72 ± 4.04	18.35 ± 2.77	21.53 ± 1.67	**<0.001 (7 < 8** <** 9<10)**	**0.048**	**0.035**	**0.003**

Mean ± SD.

^*^The *p*-value was obtained from one-way ANOVA.

^†^The *p*-value was obtained from the *post hoc* test (Bonferroni) between SS 7 and SS 8 groups.

^‡^The *p*-value was obtained from the *post hoc* test (Bonferroni) between SS 8 and SS 9 groups.

^§^The *p*-value was obtained from the *post hoc* test (Bonferroni) between SS 9 and SS 10 groups.

Boldface numbers indicate statistically significant differences at p < 0.05.

**Table 3:** Comparisons of the perfusion density among groups with a signal strength (SS) from 7 to 10.

	SS 7 (n = 99)	SS 8 (n = 82)	SS 9 (n = 42)	SS 10 (n = 36)	p-value^*^	p-value^†^	p-value^‡^	p-value^§^
Average	0.356 ± 0.049	0.375 ± 0.037	0.402 ± 0.029	0.422 ± 0.018	**<0.001 (7 < 8 < 9 = 10)**	**<0.001**	**0.001**	0.175
Superior sector	0.381 ± 0.049	0.396 ± 0.036	0.409 ± 0.034	0.422 ± 0.025	**<0.001 (7 < 8 = 9 = 10)**	**0.005**	0.399	1.000
Temporal sector	0.373 ± 0.078	0.401 ± 0.060	0.420 ± 0.051	0.399 ± 0.050	**<0.001 (7 < 8 = 9 = 10)**	**<0.001**	0.640	1.000
Inferior sector	0.389 ± 0.050	0.405 ± 0.029	0.421 ± 0.022	0.428 ± 0.025	**<0.001 (7 < 8 = 9 = 10)**	**<0.001**	0.130	1.000
Nasal sector	0.284 ± 0.073	0.315 ± 0.086	0.354 ± 0.069	0.439 ± 0.032	**<0.001 (7 < 8 < 9 < 10)**	**0.024**	**0.031**	**<0.001**

Mean ± SD.

^*^The *p*-value was obtained from one-way ANOVA.

^†^The *p*-value was obtained from the *post hoc* test (Bonferroni) between SS 7 and SS 8 groups.

^‡^The *p*-value was obtained from the *post hoc* test (Bonferroni) between SS 8 and SS 9 groups.

^§^The *p*-value was obtained from the *post hoc* test (Bonferroni) between SS 9 and SS 10 groups.

Boldface numbers indicate statistically significant differences at p < 0.05.

### Linear regression analyses between average vessel density with various ocular parameters

In univariate regression analyses, age (p = 0.036), sex (p < 0.001), IOP (p = 0.021), average RNFL thickness (p < 0.001), rim area (p < 0.001), cup/disc ratio (p < 0.001), and SS (p < 0.001) were significantly associated with average VD (Table 4). Multivariate regression analyses, which included seven significant variables from univariate analyses, showed that four factors: age (partial r = 0.133, p = 0.036), average RNFL thickness (partial r = 0.169, p = 0.008), cup/disc ratio (partial r = −0.481, p < 0.001), and SS (partial r = 0.413, p < 0.001), were significantly correlated with average VD.

**Table 4:** Linear regression analyses between average vessel density with clinical and anatomical factors.

	Univariate regression		Multivariate regression		
	β ± SE	p-value	β ± SE	Partial r	p-value
Age	0.029 ± 0.014	**0.036**	0.033 ± 0.016	**0.133**	**0.036**
Sex (0 = male, 1 = female)	0.861 ± 0.234	**<0.001**	−0.130 ± 0.246	−0.034	0.599
BCVA	−3.321 ± 1.861	0.075			
IOP	−0.076 ± 0.033	**0.021**	−0.017 ± 0.032	−0.034	0.594
Spherical equivalent	0.022 ± 0.042	0.602			
Axial length	−0.059 ± 0.084	0.485			
Central foveal thickness	0.006 ± 0.006	0.269			
Average RNFL thickness	0.054 ± 0.013	**<0.001**	0.037 ± 0.014	**0.169**	**0.008**
Rim area	3.202 ± 0.459	**<0.001**	−0.178 ± 0.501	−0.023	0.724
Cup/disc ratio	−9.126 ± 0.562	**<0.001**	−6.190 ± 0.721	−**0.481**	**<0.001**
Signal strength	0.923 ± 0.115	**<0.001**	0.733 ± 0.103	**0.413**	**<0.001**

BCVA = best-corrected visual acuity; IOP = intraocular pressure; RNFL = retinal nerve fiber layer; SE = standard error.

Boldface numbers indicate statistically significant differences at p < 0.05.

## Discussion

The present study confirmed that peripapillary microvascular density is significantly affected by SS. This trend is similar to that observed in our previous study. In brief, we previously performed 6 × 6 mm macular angiography scans and found an association between SS and macular microvascular density^26^. VD and PD values increased with as the SS increased from 7 to 9. No difference was evident between SS 9 and SS 10 in any sector, with the exception of several of the outer sectors. Venugopal *et al*.^27^ also reported that SS had a significant effect on the repeatability of OCTA with the VD values increasing in scans with higher SS values.

In this study, the VD and PD patterns observed for the four SS groups differed, depending on the sector. The superior, temporal, and inferior sectors in the SS 8, 9, and 10 groups showed no difference, but were higher than those in the SS 7 group, whereas the VD and PD in the nasal sector significantly increased as the SS rose from 7 to 10. On average, significant differences were found among the SS 7, 8, and 9 groups, but no difference was observed between the SS 9 and 10 groups, which was attributed to summing over the four sectors.

**Figure 2:**
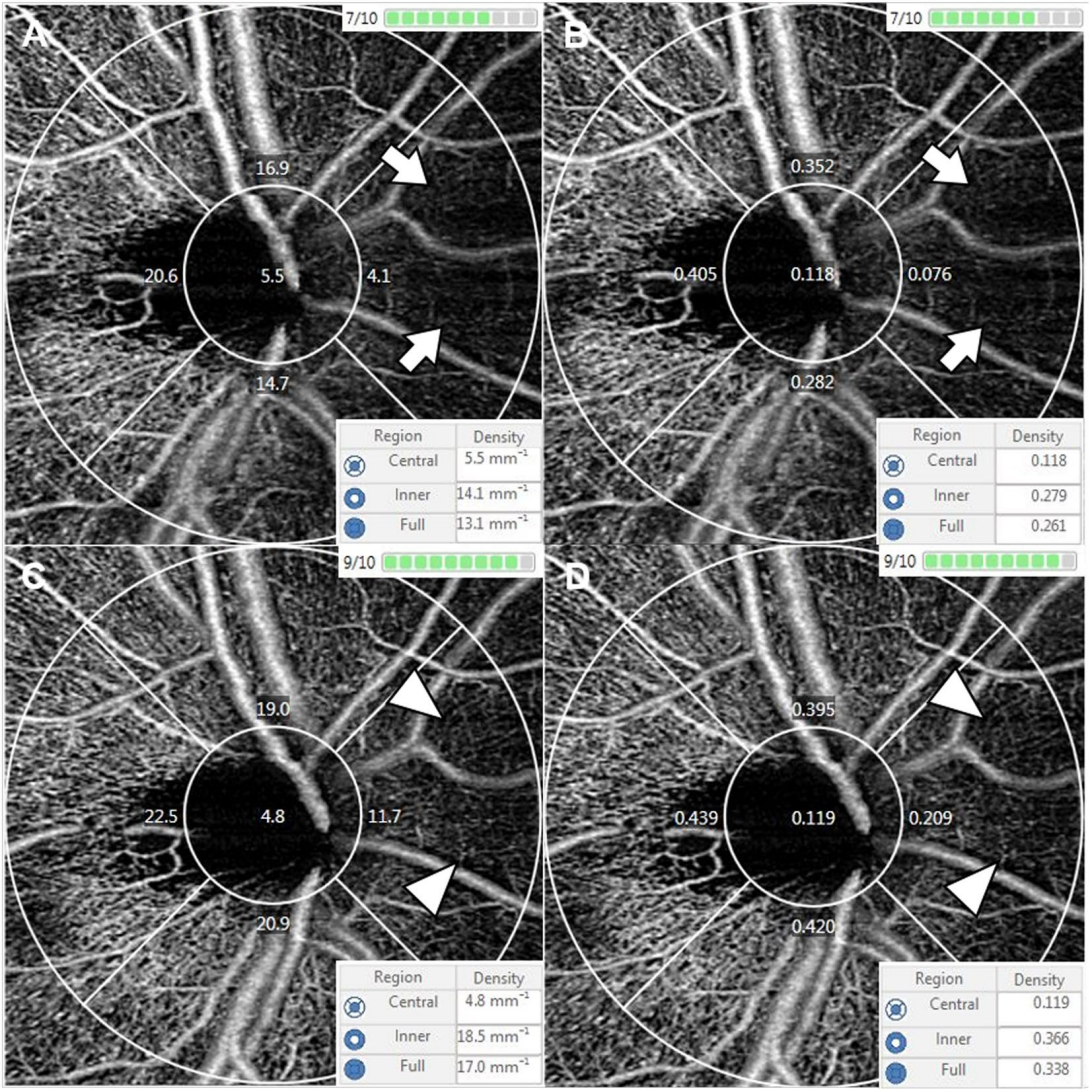
Optical coherence tomography angiography images of signal strength (SS) 7 (upper row, (**A**,**B**) and 9 (lower row, **C**,**D**) in the same subject. The average peripapillary vessel density (VD) and perfusion density (PD) of SS 7 were reduced as compared with SS 9 by 23.78% and 23.77%, respectively. Small vessels in nasal area was well detected in SS 9 (arrow head), whereas signals was significantly attenuated in SS 7 (arrow).

The results of this study differed from those of our previous study for the macular area. First, the vessel distributions in the peripapillary and macular areas were completely different. Large vessels from the optic disc, forming a retinal vessel arcade, comprised a large portion of the 3 × 3 mm angiography scan in the peripapillary area, whereas there were microvasculature and foveal avascular zones in the macular area and no large vessels. In the process of calculating the VD and PD, repeated OCT B-scans were obtained from the same location, and motion contrast was produced by calculating decorrelation/dissimilarity of repeated B-scans. To remove the invalid pixels from the OCTA image which was associated with noisy or low OCTA pixels, the thresholding algorithm was applied to the acquired unthresholded OCTA B-scans. Finally, the thresholded OCTA scan image was displayed^28^. In this process, large and small vessels may show different results, depending on the SS. Large vessels are less affected by SS, whereas signals from small vessels may be attenuated or not detected (Fig. 2)^8^. For these reasons, the results of this study differed from those of the previous study, and different patterns were observed in the four peripapillary sectors. The second reason is the difference in scanning area. In our previous study, a 6 mm macular scan was performed. The 6 × 6 mm and 3 × 3 mm scans have different scan densities and number of repetitive scans^29^.

Currently, optic disc and peripapillary perfusion OCTA analyses are used to evaluate many diseases, many of which involve glaucoma. Jia *et al*.^11^ reported that the disc flow index was reduced by 25% in the glaucoma group, and Liu *et al*.^9^ also reported decreased peripapillary VD by 13% in glaucomatous eyes (93.00% in normal eyes, 80.55% in glaucomatous eyes). Some investigators also reported that deep retinal layer and parapapillary choroidal microvasculature dropout is associated with RNFL thinning in primary open-angle glaucoma^13,19^. Other studies have reported a reduction in optic disc perfusion in glaucoma^10,12,14–18,30–32^. In addition, Wang *et al*.^33^ indicated a 12.5% reduction in the disc perfusion index of multiple sclerosis with the ON group compared to control eyes. Another study observed a significant reduction of the peripapillary VD in LHON patients (−6.2%, −10.2%, and −25.7% reduction compared to controls in the early acute stage, late-acute stage, and chronic stage, respectively)^20^. However, in most of the aforementioned studies, SS was only used as study inclusion criteria; a comparison of SS between disease and control groups was not performed. In this study, SS 7, 8, and 9 groups showed decreased values compared to the SS 10 group by 13.8%, 9.4%, and 3.8% in VD and 15.6%, 11.1%, and 4.7% in PD, respectively (Fig. 2). Although the OCTA device used in each study was different, considering the calculation process of microvascular density in OCTA as mentioned above, the effects of SS influence in the above studies cannot be excluded completely.

According to previous studies, optic disc perfusion was significantly associated with visual field defects, as well as structural measures such as the RNFL, ganglion cell-inner plexiform layer (GC-IPL), and rim area in glaucoma patients. Yarmohammadi *et al*.^32^ reported that circumpapillary VD was significantly associated with average RNFL thickness (r = 0.73) and rim area (r = 0.44), and another study found an association with RNFL (r = 0.406) and GC-IPL (r = 0.413)^34^. This trend was also observed in other studies^11,35^. In this study, age, rim area, disc area, and SS were significantly correlated with VD values, similar to previous studies. In multivariate regression analyses, the rim area was not significantly associated with average VD; however, this may be due to the interaction between average RNFL thickness and rim area. Partial r in SS was 0.413, the second highest absolute value after cup/disc ratio (r = −0.481). Thus, SS plays a significant role and should be considered, along with the well-known structural factors associated with glaucoma.

Scan image quality is essential for the quantitative assessment of microvascular density. Various factors such as patient cooperation, media opacity, and operator skill can affect the image quality of OCT scan. OCTA images with a higher SS show better clarity and improved segmentation error, which can increases the reliability of the measurement. Additionally, previous studies have reported that quantitative metrics in the OCTA is significantly correlated with the SS^36,37^. If OCTA scan image is obtained with low SS, the repeatability and reproducibility are reduced, and the OCTA measurements become unreliable. For accurate data analyses, recommended SS values which is defined by the manufacturer’s proprietary guidelines should be obtained. For example, Cirrus HD-OCT recommends ≥6 (signal strength, 0 to 10), Spectralis OCT (Heidelberg Engineering, Heidelberg, Germany) recommends more than 15 (Q score, 0 to 40), and RTVue (Optovue, Fremont, CA, USA) recommends ≥45 (signal strength index, 0 to 100) for macular patterns and ≥35 for retinal scanning^38^. However, there has still been no discussion of the optimal SS level in OCTA analyses. All subjects enrolled in the present study were healthy, young volunteers who did not have any ocular or systemic abnormalities. In addition, the four groups were compared under the same conditions (e.g., age, sex, and various ocular factors). Therefore, the effects of SS on the peripapillary microvascular density are considered a meaningful finding, and the method of microvascular density estimation in various articles previously reported should be considered in correlation with SS.

This study had several limitations. First, the effects of SS were not verified on various OCTA devices. Although the OCTA measurement method is similar in that it uses the decorrelation of moving particles in vessels, the commercial OCTA devices currently used are based on different imaging algorithms; thus, the effects of SS on measured values may vary from one device to another. Second, the effects of SS were examined in normal healthy subjects; thus, its effects in diseased eyes are unknown. Third, the numbers of SS 9 and 10 groups were slightly lower than those of SS 7 and 8 groups. Finally, to investigate the microvasculature of the optic nerve head, it may be helpful to exclude large vessels using various methods such as manual segmentation or applying a specific threshold algorithm, but we did not perform that. Since it is considered that modifications of scan image would not be useful in clinical practice, we used built-in software only. Additional studies are needed to overcome these limitations.

In conclusion, we investigated the effect of SS on the OCTA measurements in the young healthy subjects and found that peripapillary VD has a significant correlation with SS. Therefore, when analyzing average VD and PD, it is necessary to try to obtain the highest SS possible. Considering our results, an SS of at least 9 in the average sector and 8 in superior, temporal, and inferior sectors may be needed in the analysis of peripapillary microvascular density. SS is a significant factor that affects peripapillary microvascular density, and its influence is similar to the well-known structural parameters of glaucoma. Our study is the first to analyze the effects of SS on peripapillary VD. The results from this study are expected to be particularly useful to evaluate peripapillary perfusion, and clinicians should consider SS when interpreting optic disc OCTA scans.

## Methods

This was a prospective cohort study. The study protocol was approved by the Armed Forces Medical Command Institutional Review Board (Seongnam, Republic of Korea) and followed to the tenets of the Declaration of Helsinki. All subjects who met the eligibility criteria provided written informed consent to participate.

### Subjects

This study initially included 588 eyes from 294 healthy, young volunteers who visited the Armed Forces Capital Hospital for a health screening checkup. All subjects underwent comprehensive ophthalmic examinations including a slit-lamp biomicroscopy, refraction,best-corrected visual acuity (BCVA), intraocular pressure (IOP). Additionally, central corneal thickness (NT-530, NIDEK Co., LTD, Gamagori, Aichi, Japan), axial length (IOLMaster, Carl Zeiss Meditec, Dublin, CA, USA) were measured, and OCT and OCTA using the Zeiss Cirrus 5000 (Carl Zeiss Meditec, Dublin, CA, USA) was performed. All subjects underwent a 3 × 3 mm angiography scan in the peripapillary area using the AngioPlex OCTA device from the Zeiss Cirrus 5000 system, without pupil dilation, and no eyedrops or gels were used to degrade the image quality. The excluded subjects were those with a history of systemic disease including diabetes and hypertension, a history of neuro-ophthalmic or retinal disease, media opacity, glaucoma, a history of ocular trauma, an IOP > 21 mmHg, BCVA < 20/25, OCTA scan with an SS < 7, a, and the presence of a segmentation error in the OCTA scan. If both eyes met the inclusion criteria, one eye was randomly selected. Finally, 259 eyes from 259 healthy, young subjects were included in this study.

### OCT and OCTA

**Figure 3:**
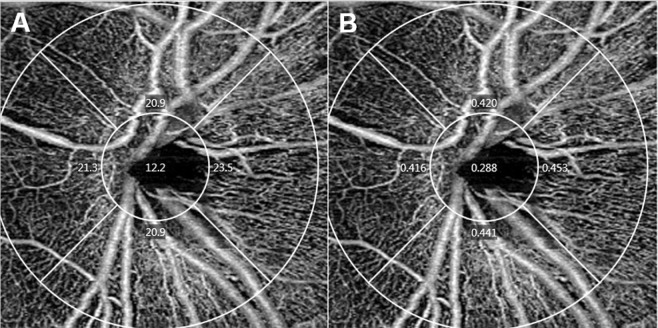
A representative optical coherence tomography angiography (OCTA) image of a 27-year-old healthy male subject in the signal strength (SS) 10 group. Vessel density (VD; **A**) and perfusion density (PD; **B**) map in 3 × 3 mm scan consisting of a centered 1 mm diameter circle and four outer sectors including superior, nasal, inferior, and temporal areas. The analysis of peripapillary microvascular density included measurements from the four sectors. Average measurements were calculated, and the center 1 mm area was excluded. The average VD was 21.6 mm^−1^ and the average PD was 0.433.

The Zeiss HD-OCT 5000 instrument with an AngioPlex OCTA device was used to acquire microvasculature images of the peripapillary area. This instrument operated at a central wavelength of 840 nm and a speed of 68,000 A-scans per second. In the 3 × 3 mm scan pattern, there were 245 A-scans in each B-scan along the horizontal dimension and 245 B-scan positions along the vertical dimension. Each B-scan was repeated four times at the same position^29^. The optical microangiography-complex algorithm analyzed the change in complex signals (both intensity and phase changes contained within sequential B-scans performed at the same position)^39,40^, and then generated en face microvascular images. The vascular images of the superficial capillary plexus (SCP), which spanned from the internal limiting membrane to the inner plexiform layer, and the deep capillary plexus, which extended from the inner nuclear layer to the outer plexiform layer, were displayed separately. The AngioPlex incorporated the FastTrac retinal tracking technology to reduce motion artifacts. All scans were analyzed using Cirrus OCTA software (AngioPlex software, version 10.0; Carl Zeiss Meditec). VD (defined as the total length of the perfused vasculature per unit area in the region of measurement) and PD (defined as the total area of the perfused vasculature per unit area in the region of measurement) of the SCP in the 3 × 3 mm scan were measured automatically^41^; the scan consisted of a centered 1 mm diameter circle and four outer sectors including superior, nasal, inferior, and temporal areas (Fig. 3A,B). All scans were performed by the same experienced examiner and reviewed individually by two investigators (H.B.L. and Y.W.K.) for quality evaluation (i.e., SS, segmentation error, loss of fixation, and motion artifacts), and substandard scans were excluded. The central foveal thickness (CFT), retinal nerve fiber layer (RNFL) thickness, and optic disc parameters (including the rim area and cup/disc ratio) were measured using a macular cube 512 × 128 combination scan and an optic disc cube 200 × 200 scan.

### Statistical analyses

Subjects were divided into four groups according to the SS of the angiography scan: SS 7, 8, 9, and 10. The continuous variables among the four groups were compared by one-way analysis of variance (ANOVA) with a *post-hoc* Bonferroni correction, and the categorical variables were compared using the χ2 test. The VD and PD in the peripapillary area were also compared among the four groups using one-way ANOVA with a *post hoc* Bonferroni correction. Univariate and multivariate linear regression analyses were performed to identify and evaluate the clinical factors associated with average peripapillary VD. All statistical analyses were performed using SPSS statistical software for Windows, version 21.0 (SPSS, IBM Corp, Armonk, NY, USA) and GraphPad Prism^TM^ (version 6.0; GraphPad Software, San Diego, CA, USA). Snellen BCVA results were converted into the logarithm of the minimum angle of resolution value. Continuous variables are presented as the mean ± standard deviation. Differences were considered significant at p < 0.05.

## Data Availability

Data supporting the findings of the current study are available from the corresponding author on reasonable request.
